# SWIFT: A deep learning approach to prediction of hypoxemic events in critically-Ill patients using SpO_2_ waveform prediction

**DOI:** 10.1371/journal.pcbi.1009712

**Published:** 2021-12-21

**Authors:** Akshaya V. Annapragada, Joseph L. Greenstein, Sanjukta N. Bose, Bradford D. Winters, Sridevi V. Sarma, Raimond L. Winslow

**Affiliations:** 1 Johns Hopkins University School of Medicine, Baltimore, Maryland, United States of America; 2 Institute for Computational Medicine, The Johns Hopkins University, Baltimore, Maryland, United States of America; 3 Department of Electrical and Computer Engineering, The Johns Hopkins University, Baltimore, Maryland, United States of America; 4 Anesthesiology and Critical Care Medicine, Johns Hopkins University School of Medicine, Baltimore, Maryland, United States of America; 5 Department of Biomedical Engineering, The Johns Hopkins University School of Medicine & Whiting School of Engineering, Baltimore, Maryland, United States of America; University of Washington, UNITED STATES

## Abstract

Hypoxemia is a significant driver of mortality and poor clinical outcomes in conditions such as brain injury and cardiac arrest in critically ill patients, including COVID-19 patients. Given the host of negative clinical outcomes attributed to hypoxemia, identifying patients likely to experience hypoxemia would offer valuable opportunities for early and thus more effective intervention. We present SWIFT (SpO_2_ Waveform ICU Forecasting Technique), a deep learning model that predicts blood oxygen saturation (SpO_2_) waveforms 5 and 30 minutes in the future using only prior SpO_2_ values as inputs. When tested on novel data, SWIFT predicts more than 80% and 60% of hypoxemic events in critically ill and COVID-19 patients, respectively. SWIFT also predicts SpO_2_ waveforms with average MSE below .0007. SWIFT predicts both occurrence and magnitude of potential hypoxemic events 30 minutes in the future, allowing it to be used to inform clinical interventions, patient triaging, and optimal resource allocation. SWIFT may be used in clinical decision support systems to inform the management of critically ill patients during the COVID-19 pandemic and beyond.

## Author summary

Hypoxemia, or loss of blood oxygen saturation, is a dangerous condition that drives morbidity and mortality in critically ill patients, including COVID-19 patients and patients with brain injury or cardiac arrest. The ability to identify hypoxemia before it occurs would expand the possibilities for effective clinical interventions. To this end, we present SWIFT (SpO_2_ Waveform ICU Forecasting Technique), a deep learning model that can predict blood oxygen saturation 5 and 30 minutes in the future in critically ill patients. In testing, SWIFT identified more than 80% and 60% of hypoxemic events in critically ill and COVID-19 patients, respectively. SWIFT can predict both the occurrence and magnitude of hypoxemic events, which provides clinical information that can help prevent hypoxemia in critically ill patients. SWIFT can be used in clinical decision support systems to improve the management of patients at risk for hypoxemia during the COVID-19 pandemic and beyond.

## Introduction

Hypoxemia, or a decrease in blood oxygen saturation, is a common symptom in critically ill patients, with a multinational, multicenter study finding that hypoxemia is a significant risk factor for mortality, with prevalence greater than 50% in ICU patients [1]. Severe hypoxemia can cause permanent brain injury, end-organ shock and cardiac arrest, and even mild or moderate hypoxemia contributes to increased mortality risk by decreasing resistance to infection and wound healing [2].

Severe cases of COVID-19 are also characterized by hypoxemia and dyspnea (difficulty breathing) which can rapidly progress to respiratory failure [3]. These patients often require advanced life support measures including invasive mechanical ventilation, hospitalization in ICUs and even extra-corporeal membrane oxygenation (ECMO). During the COVID-19 pandemic, ventilators and ICU beds have become scarce resources with insufficient capacity in the hardest hit regions [4]. As the COVID-19 pandemic continues to exact a heavy mortality toll with over half a million deaths directly attributed to the disease in the United States alone and herd immunity by vaccination remains elusive, it is important to find ways to manage these scarce resources and identify patients unable to maintain oxygen saturation without intervention. Clinically, an important decision point in the management of COVID-19 patients is determining whether the patient requires endotracheal intubation, a form of invasive ventilation [3]. Triage systems using monitoring of blood oxygenation to inform life support measures are tremendously useful for directing resource allocation and have been demonstrated to reduce mortality [5].

Given the host of negative clinical outcomes attributed to hypoxemia, identifying patients likely to experience acute hypoxemia in the near future would offer valuable opportunities for rapid intervention. Life support interventions ranging from supplemental oxygen to invasive ventilation prior to the onset of hypoxemia can mitigate or prevent the morbidity and mortality associated with hypoxemia [2]. Moreover, identifying patients not at imminent risk of hypoxemia represents an opportunity to conserve ventilators and ICU beds in the context of resource shortages arising from a global pandemic.

To this end, we present SWIFT (SpO_2_ Waveform ICU Forecasting Technique), a neural network that predicts the blood oxygen saturation (SpO_2_) waveform for critically ill patients, 5 and 30 minutes in the future. SWIFT is unique for several reasons. First, SWIFT predicts both the occurrence and magnitude of hypoxemic events, and its prediction time horizon provides enough time for potential clinical interventions prior to acute desaturation events. Prior studies have made predictions on short time horizons (20 seconds to 5 minutes), leaving little room for potential clinical interventions [6–9]. Moreover, most other attempts at hypoxemia prediction predict only a class value (hypoxemia vs. no hypoxemia, or mild hypoxemia vs. severe hypoxemia vs. no hypoxemia) rather than an actual SpO_2_ value [6–8]. Clinically, there is a large difference between a transient dip in SpO_2_ to 91% versus an acute desaturation to 75% SpO_2_, though both would be considered hypoxemia. SWIFT recognizes this difference, hence providing important clinical information.

Second, SWIFT employs a Long Short-Term Memory (LSTM) architecture with only prior SpO_2_ values as inputs, hence allowing SWIFT to make predictions with limited, routinely acquired and readily available data. LSTM models are a type of recurrent neural network well-suited to modeling of time-series data that have shown promise in clinical applications [10–12]. One prior study did use LSTM architectures with prior SpO_2_ values as inputs, but this model was limited to classification of timepoints as either hypoxemic or not with a 5 minute time horizon, and the total ROC-AUC was less than 0.75 [7]. In contrast, other SpO_2_ prediction models have used complex, multifactorial data requiring extensive monitoring of patient vitals, demographic data, or ventilator settings [8,9]. This limits their utility to only those patients for whom all of this data is readily available.

Third, SWIFT predicts more than 80% of all hypoxemic events (sensitivity) with positive predictive value (PPV) above 94% in two test-sets of ventilated and non-ventilated critically ill patients, and more than 60% of all hypoxemic events with PPV above 98% in a test-set of COVID-19 patients, across all timepoints for both the 5 minute and 30 minute time horizons. SWIFT also provides waveform predictions with an average mean squared error less than .0007 across all patient-stays. These results represent a marked improvement over recently published prediction algorithms. Auto-regressive models with PPV >90% have been limited to prediction time horizons less than 60 seconds [6], and ensemble-based machine learning models to classify hypoxemic events 5 minutes in the future were estimated to capture only 30% of hypoxemic events [9]. To our knowledge, no other study has demonstrated waveform prediction.

Finally, SWIFT is highly generalizable across hospital systems, timeframes, and patient conditions. Though trained on only patients without COVID-19, it performs comparably on patients who received mechanical ventilation during their ICU stay and those who did not, and patients with and without a COVID-19 diagnosis. Other studies have been limited to specific groups such as pediatric patients on mechanical ventilation [8], orthopedic postoperative adult patients [6], or patients undergoing surgery in the operating room [7–9].

## Results

SWIFT is effective at predicting hypoxemia events (both occurrence and magnitude) across a variety of patient populations (Ventilated patients, non-ventilated patients, patients with COVID-19 diagnosis, critically ill patients without COVID-19 diagnosis). We show this through performance evaluation of SWIFT for prediction of hypoxemia at individual timepoints 5 and 30 minutes into the future, and evaluation of SWIFT for direct SpO_2_ waveform forecasting.

### SWIFT overview

**Fig 1:**
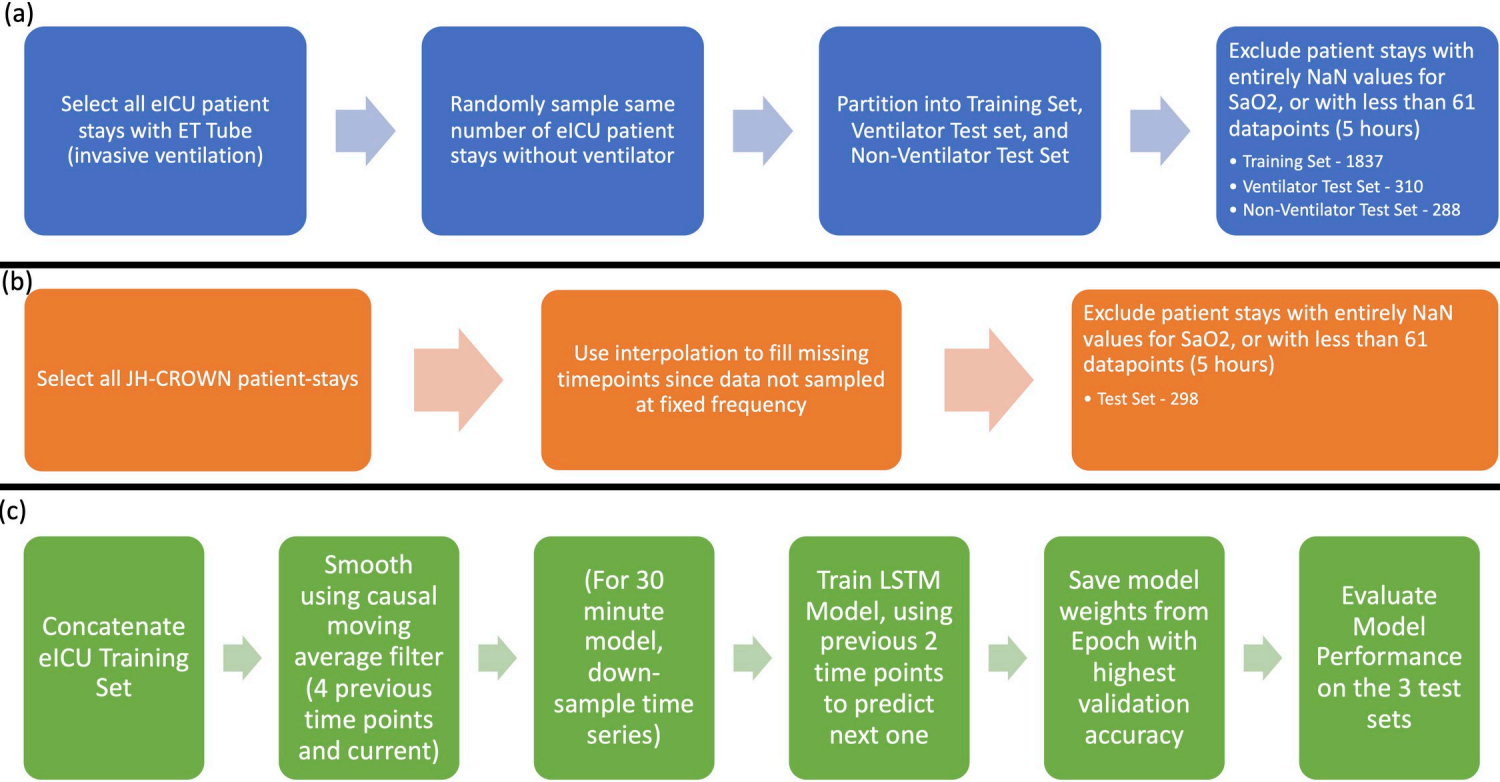
Schematic illustrating data processing for (a) eICU patients and (b) JH-CROWN patients, and (c) model training and testing pipelines.

SWIFT utilizes an LSTM (Long Short-Term Memory) neural network architecture trained on the SpO_2_ waveforms from critically ill patients in the eICU database [13] (Fig 1A). The eICU database contains patients admitted to intensive care units across 208 United States hospitals in 2014 and 2015. A similar data processing procedure was used to obtain another, distinct test-set of patients from the Johns Hopkins JH-CROWN database, where all patients had COVID-19 (Fig 1B). We trained two different models, one which predicts hypoxemia 5 minutes in the future (SWIFT-5) and one which predicts hypoxemia 30 minutes in the future (SWIFT-30) (Fig 1C). Both models take the two previous timepoints of data (10 minutes prior data for SWIFT-5, 60 minutes prior data for SWIFT-30) as inputs and predict SpO_2_ value for the next time point. We used these predictions to forecast the exact SpO_2_ waveform, and to classify individual timepoints as hypoxic events based on a threshold of 92% SpO_2_.

We considered 92% SpO_2_ to be the threshold for hypoxemia, as SpO_2_ below 92% has been shown to be associated with adverse events in a broad population of outpatient adults with pneumonia [14], and is the low end of the National Institutes of Health’s recommended SpO_2_ target range for COVID-19 patients [15]. Moreover, 92% SpO_2_ is between the World Health Organization’s hypoxemia treatment threshold (94%) and clinical emergency threshold (90%) [16], and has been used as the hypoxemia cut-off in other prediction studies [8,9].

We demonstrate the SpO_2_ waveform and hypoxemia prediction capabilities of SWIFT on three different test sets of patient-stays: patient-stays with and without the use of invasive ventilation at any point in the stay from the eICU database, and a test set of critically ill COVID-19 patients from the JH-CROWN database. While the eICU database consists of critically ill patients with a variety of diagnoses, the JH-CROWN database consists of patients specifically diagnosed with and admitted to hospital for COVID-19 at a single academic center and its affiliated hospitals in 2020.

We hypothesized that patients requiring invasive ventilation at some point in their ICU stay would have different profiles with respect to hypoxemia than those never requiring invasive ventilation. A chi-squared test of independence between use of mechanical ventilation and number of hypoxemic time-points in the dataset was statistically significant for all groups (eICU 30 minute time-series, p = 1.4e-303; eICU 5 minute time-series, p = 0.0), hence motivating our treatment of eICU patient-stays utilizing invasive ventilation at any point as a distinct test set from those patient-stays never utilizing invasive ventilation. We did not split the JH-CROWN test-set into ventilator and non-ventilator test-sets since all patients had the same diagnosis and the overwhelming majority were on ventilators, in contrast to the patients in the eICU database. Importantly, the different patient populations studied vary dramatically with respect to frequency of hypoxic events, duration of available time-series data, and reason for hospital admission (Table 1), yet our models are effective at recapitulating the patient’s SpO_2_ waveform over time and predicting hypoxemic events across these diverse patient populations.

**Table 1:** Summary of patient characteristics used in model testing.

Characteristics	eICU Ventilator– 30 minutes	eICU non-Ventilator– 30 minutes	JH CROWN– 30 minutes	eICU Ventilator– 5 minutes	eICU non-Ventilator– 5 minutes	JH CROWN– 5 minutes
**# Patient Stays**	310	288	298	310	288	298
**Median Hypoxic events/patient stay**	9	1	79.5	55	4	481
**Number patient-stays with No Hypoxic events**	64	138	10	53	123	10
**Number patient-stays with all Hypoxic events**	4	1	1	3	0	1
**Median number of timepoints per patient-stay**	133	79.5	1083	798	477	6497
**Median Age at patient-stay**	62.0 (on admission)	65.0 (on admission)	60.98 (on Dec 15, 2020 assuming 365 days/year)	62.0 (on admission)	65.0 (on admission)	60.98 (on Dec 15, 2020 assuming 365 days/year)
**% patient-stays with Female patients**	42.9%	49.3%	41.2%	42.9%	49.3%	41.2%
**% patient-stays with White/Caucasian patients**	81.3%	76.7%	26.2%	81.3%	76.7%	26.2%
**Admissions Diagnosis**	Top 3: (1) CABG alone, coronary artery bypass grafting (42 stays) (2) Cardiac arrest (24 stays) (3) Emphysema/bronchitis (15 stays)	Top 3: (1) Not recorded (15 stays) (2) Sepsis, pulmonary (14 stays) (3) Rhythm disturbance (13 stays)	All patients with COVID-19	Top 3: (1) CABG alone, coronary artery bypass grafting (42 stays) (2) Cardiac arrest (24 stays) (3) Emphysema/bronchitis (15 stays)	Top 3: (1) Not recorded (15 stays) (2) Sepsis, pulmonary (14 stays) (3) Rhythm disturbance (13 stays)	All patients with COVID-19

(Note: Demographic information on age, gender, and race only available for 294 of 298 patients in the JH-CROWN dataset)

### SWIFT effectively predicts hypoxic events 5 and 30 minutes in the future

**Fig 2:**
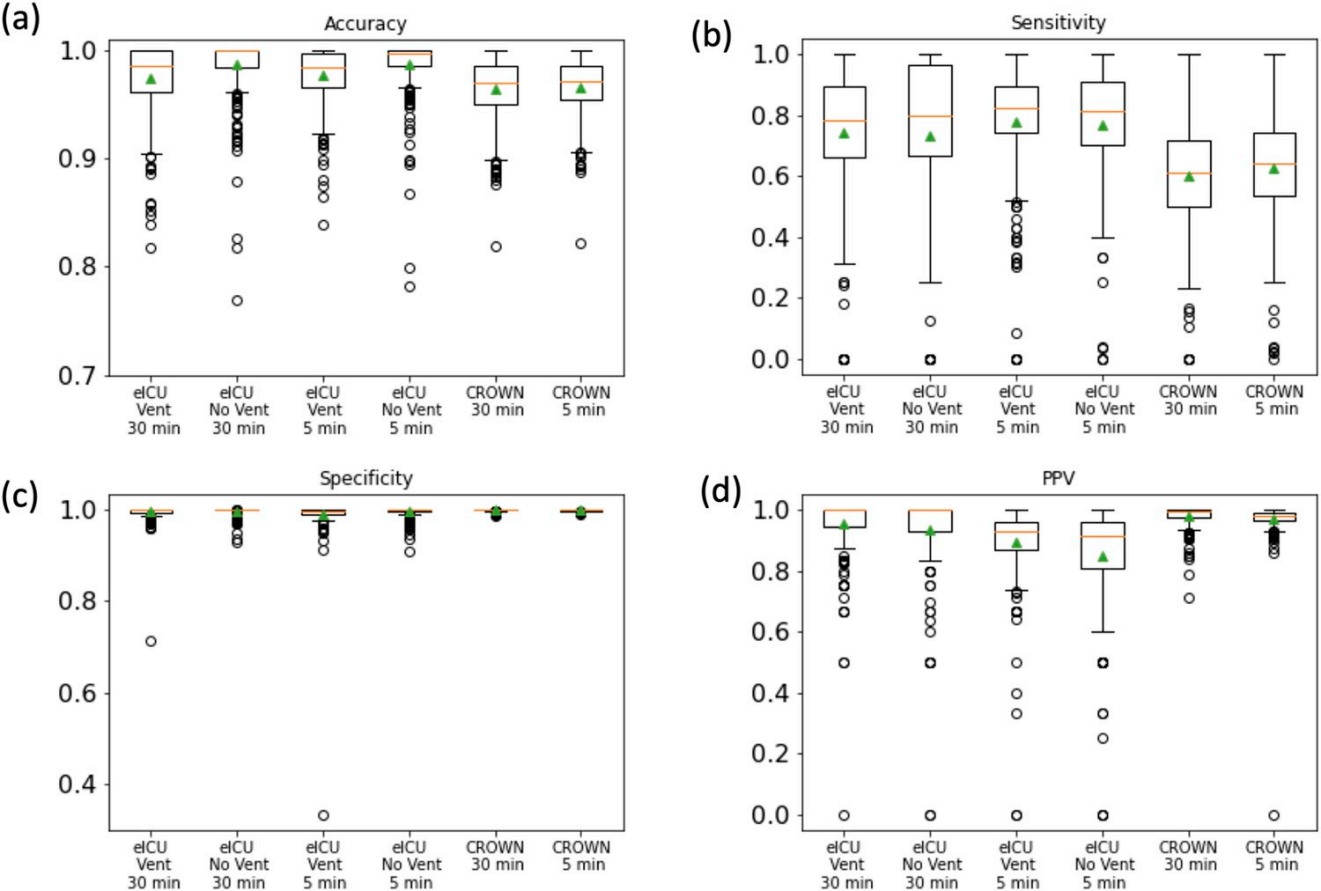
**(a) Accuracy, (b) Sensitivity, (c) Specificity, and (d) PPV for SWIFT-5 and SWIFT-30 tested on eICU patients with and without ventilators, and JH-CROWN patients with COVID-19.** In each box-and-whisker plot, the individual datapoints come from evaluation of the model on each of the test-set patients. The box extends from Q1 to Q3. The orange line represents the median value and the green triangle represents the mean value. The upper whisker extends to the highest value below Q3+1.5*(Q3-Q1), and the lower whisker extends to the lowest value below Q1-1.5*(Q3-Q1). Points beyond the whiskers are considered outliers.

We used SWIFT on the SpO_2_ time-series of each patient-stay in the three test sets to predict, based on the prior two time points, whether each time-point was expected to be a hypoxemic event or not. Averaging across the value for each patient-stay, SWIFT-5 and SWIFT-30 both achieved mean accuracy greater than 96% for both the eICU and JH-CROWN patient-stays (Fig 2A), mean sensitivity greater than 73% for eICU patient-stays and greater than 59% for JH-CROWN patient-stays (Fig 2B), mean specificity greater than 99% for both the eICU and JH-CROWN patient-stays (Fig 2C) and mean PPV greater than 84% for eICU-patient stays and greater than 97% for JH-CROWN patient-stays (Fig 2D). The quality of predictions made for COVID patients is comparable to that of the predictions made on patients without COVID, which is notable given that SWIFT was trained exclusively on non-COVID patients. Interestingly, SWIFT-30 demonstrates performance comparable to SWIFT-5, despite the much larger prediction time horizon.

While there are a few patient-stays for whom sensitivity and PPV are low (<50%), by and large these models predict hypoxemic events effectively. In each test-set, for patients for whom sensitivity could be calculated (atleast one hypoxemic event occurred), patients with sensitivity less than 0.5 had significantly different mean number of hypoxemic events than patients with sensitivity greater than or equal to .5 (S1 Table, Welch’s t-test for identical means, p < 1e-4 for all test-sets). The median number of hypoxemic events was also lower among patients with sensitivity less than .5 for each test-set (S1 Table), This illustrates that patients for whom SWIFT’s sensitivity was low also tended to be those with less hypoxemic timepoints overall.

**Fig 3:**
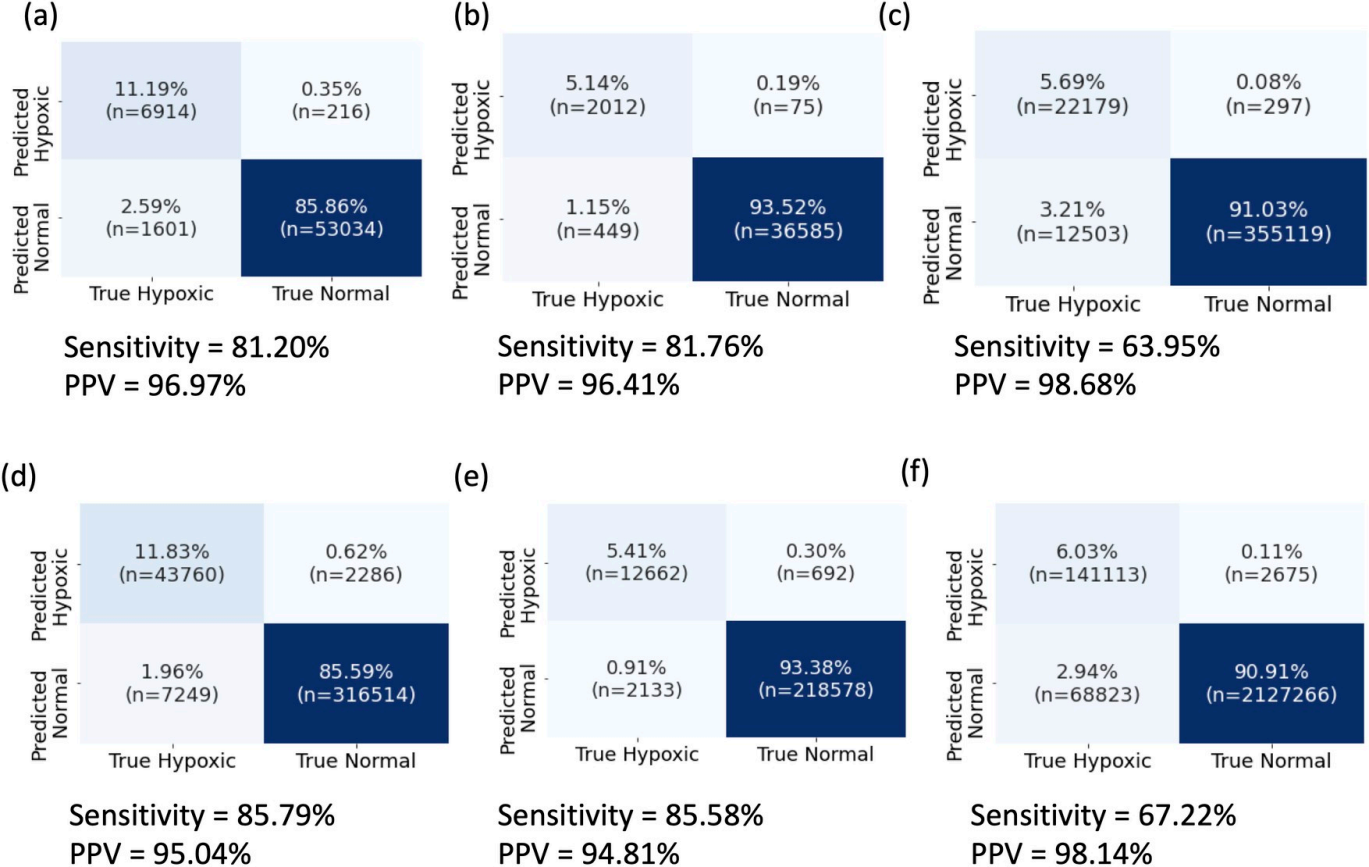
Confusion matrices aggregated across all timepoints for all patients for (a) eICU Ventilator– 30 minute, (b) eICU No ventilator– 30 minute, (c) JH-CROWN– 30 minute, (d) eICU Ventilator– 5 minute, (e) eICU No ventilator– 5 minute, (f) JH-CROWN– 5 minute.

Moreover, when all timepoints are aggregated across patient-stays, SWIFT classifies hypoxic events with remarkable success (Fig 3). Across all time-points in all test-sets for both SWIFT-5 and SWIFT-30 the False Positive rate is less than 0.65% and the False Negative Rate is less than 4%. While the datasets are imbalanced with far less hypoxic timepoints than non-hypoxic events, the high PPV and sensitivity demonstrate that SWIFT accurately classifies hypoxic timepoints. Both SWIFT-5 and SWIFT-30 represent a substantial improvement over the current state-of-the art method, Prescience, which uses machine learning to predict hypoxic events in a 5 minute window, with a binary classifier [9]. Prescience predicts 44% of hypoxic events (recall or sensitivity) with a precision (or PPV) of 30%. For a PPV of 70%, Prescience’s sensitivity falls to 9%. In contrast, SWIFT uses a waveform forecasting approach to achieve PPV above 94% and sensitivity above 80% across all timepoints in each of the eICU test sets (ventilated and non-ventilated), and PPV above 98% and sensitivity above 60% across all timepoints in the JH-CROWN test set. We also tested SWIFT models trained with 1, 3, 4 or 5 prior SPO2 inputs, and in aggregate these models had nearly identical aggregate specificity, with some improvements in sensitivity for increased numbers of inputs (S2 Fig). We chose to focus on models with 2 prior inputs as they exhibited strong performance with a limited amount of input data required (10 minutes for SWIFT-5, 60 minutes for SWIFT-30).

### SWIFT effectively predicts the SpO_2_ waveform for individual patients

**Fig 4:**
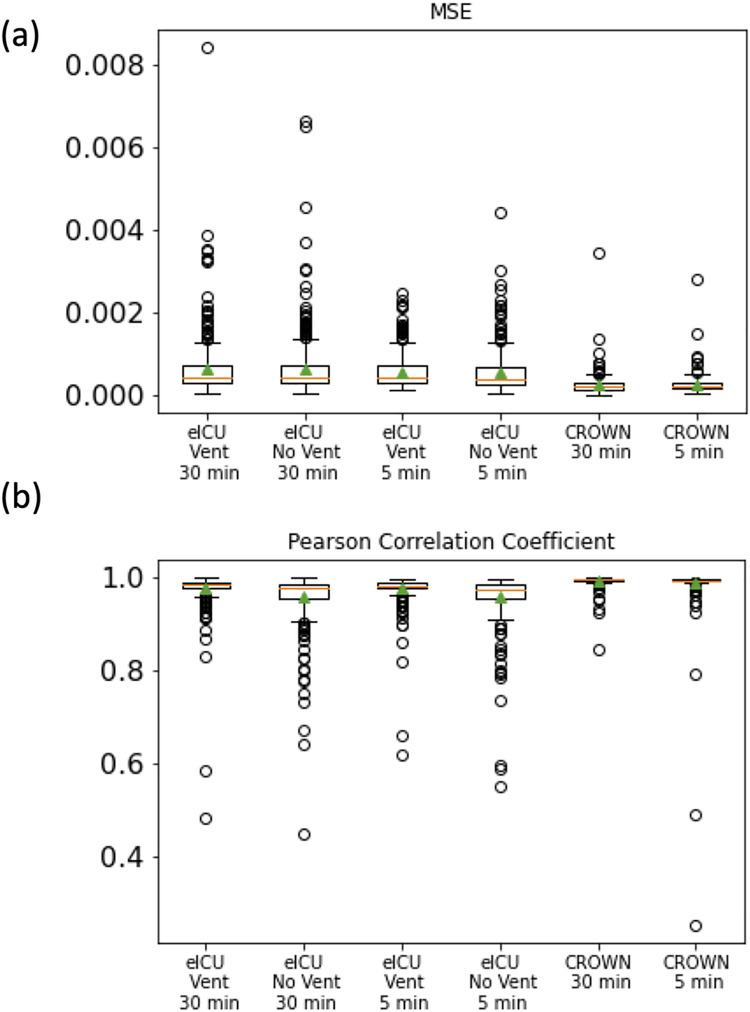
**(a) Mean-Squared Error and (b) Pearson Correlation Coefficient for waveform predictions for eICU and JH-CROWN patients.** In each box-and-whisker plot, the box extends from Q1 to Q3. The orange line represents the median value and the green triangle represents the mean value. The upper whisker extends to the highest value below Q3+1.5*(Q3-Q1), and the lower whisker extends to the lowest value below Q1-1.5*(Q3-Q1). Points beyond the whiskers are considered outliers.

**Fig 5:**
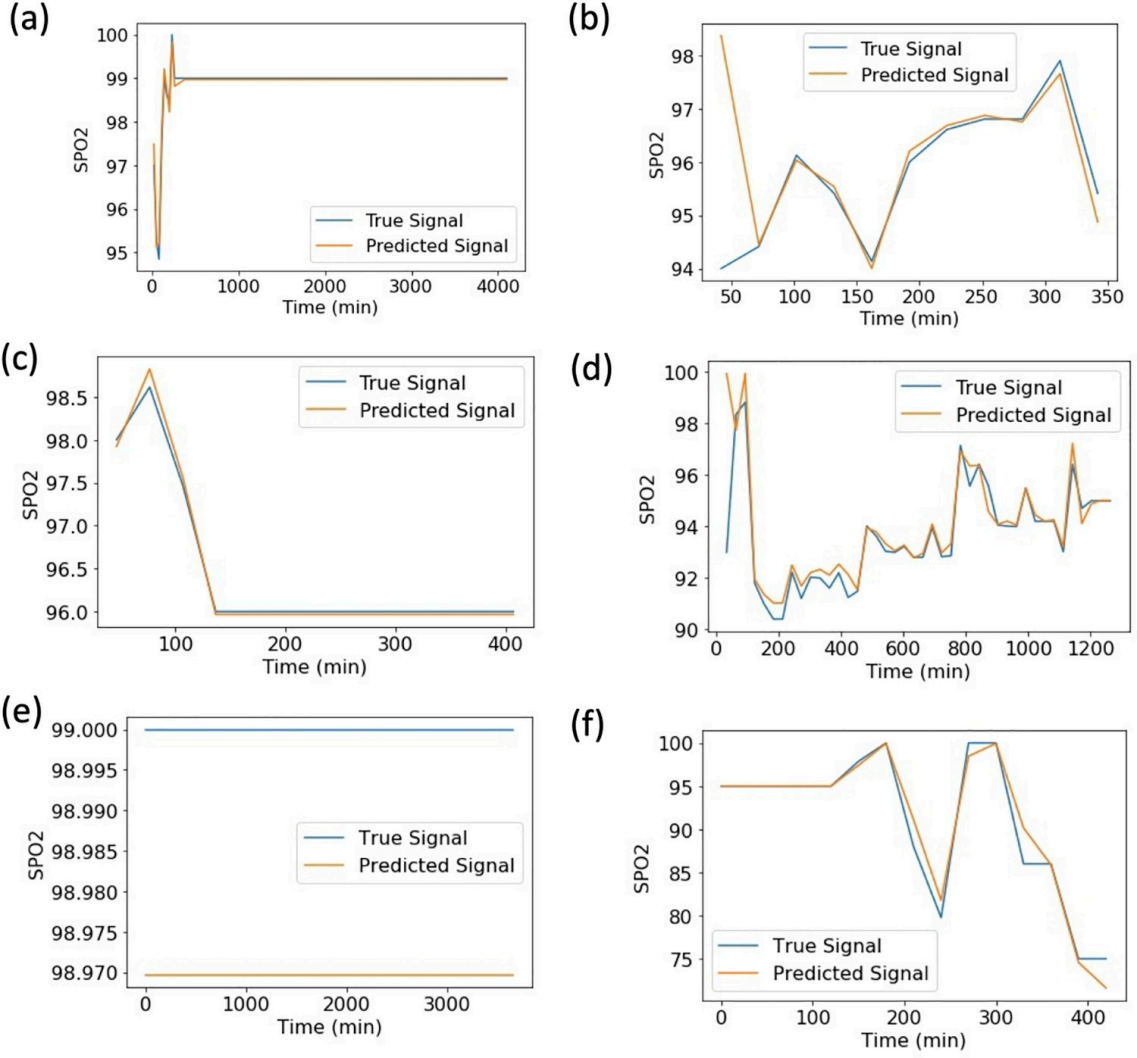
Examples of the best and worst fits by MSE for SWIFT-30 model tested on (a) eICU Ventilated patients–best fit, (b) eICU Ventilated patients–worst fit, (c) eICU Non-Ventilated patients–best fit, (d) eICU Non-Ventilated patients–worst fit, (e) JH-CROWN patients–best fit, (f) JH-CROWN patients–worst fit.

We used SWIFT to predict the SpO_2_ time-series of each patient-stay in the three test sets in order to determine the magnitude of potential hypoxic events and the overall time-evolution of transient hypoxic events. On average across all patient-stays, SWIFT-5 and SWIFT-30 both achieved mean-squared-error (MSE) from the true patient time-series of less than .0007 (Fig 4A), and a Pearson correlation coefficient with the true patient time-series of greater than .95 (Fig 4B). This indicates that the waveform predictions used to predict hypoxic events are extremely close to the true waveforms. Fig 5 shows examples of SWIFT-30’s best and worst by waveform predictions by MSE (S1 Fig contains the same information for SWIFT-5), qualitatively demonstrating the accuracy with which SWIFT recapitulates SpO_2_ waveform.

This waveform prediction allows for the forecasting of the magnitude of hypoxic events rather than their occurrence alone. This may have implications for patient management, especially in the context of limited ICU beds and shortages of ventilation machinery during the COVID-19 pandemic [4].

## Discussion

SWIFT is a Long Short-Term Memory neural network model capable of predicting the magnitude and occurrence of hypoxemic events 5 and 30 minutes in the future, using only prior SpO_2_ values. We tested SWIFT on three different test sets of ICU patient-stays, including patients both requiring and not requiring mechanical ventilation during their ICU stay, and patients with and without COVID-19. Across all time points in these test-sets, SWIFT predicts more than 80% of all hypoxemic events (sensitivity) with PPV above 94% in test-sets of critically ill patients, and more than 60% of all hypoxemic events with PPV above 98% in test-sets of COVID-19 patients, for both the 5 minute and 30 minute time horizons. Additionally, SWIFT-5 and SWIFT-30 accurately predicted SpO_2_ waveforms for each patient-stay with an average MSE below .0007 and an average Pearson’s correlation coefficient greater than .95.

SWIFT may be especially useful in the context of the COVID-19 pandemic or future similar pandemics with high numbers of patients experiencing hypoxemia and limited supplies of ventilators and ICU beds. Strategies to reduce the demand for mechanical ventilation have been identified as a priority for resource management during the pandemic [4]. To this end, SWIFT can help identify patients likely to experience imminent hypoxemic events versus patients likely to remain stable and offer insights into the magnitude of the potential hypoxemic event. This can enable the increased management of patients off of ventilators, and if needed, offer another data point to be used in the triaging of patients for therapy.

Beyond the COVID-19 pandemic, SWIFT could be easily deployed in real time, in low-resource settings without access to complex clinical informatics or large amounts of memory storage. Since SWIFT’s only model inputs are two previous values of SpO_2_, the barriers to use are minimal. SpO_2_ can be assessed using simple, non-invasive pulse oximeters. Pulse oximetry is nearly ubiquitous in hospitals and critical care units in the developed world, and substantial effort has been dedicated to increasing the use of pulse oximetry in low resource settings [17]. Given the existing need for hypoxemia monitoring in low and middle income countries and challenges in access to oxygen therapy, SWIFT’s predictive capabilities can play a crucial role in identifying patients likely to experience hypoxemic events and in informing resource allocation decisions [18]. Specifically, since SWIFT-30 uses data sampled at 30 minute intervals (rather than 5 minute intervals), it is especially suitable for scenarios in which high frequency monitoring is not available.

Moreover, SWIFT provides benefits by waveform prediction of SpO_2_ rather than only binary classification of events as provided by other existing models [7–9]. Studies have demonstrated that pulse oximetry has high levels of false alarms, often for clinically insignificant reasons such as patient movement or skin condition, which can contribute to alarm fatigue [19,20]. Alarm fatigue may lead to slower or absent responses to truly dangerous events [21]. Since SWIFT provides a prediction of SpO_2_ magnitude as much as 30 minutes in the future, minor anticipated hypoxemic events can be distinguished from more severe ones, with sufficient time horizon to allow for decision making on this basis. While SWIFT cannot correct for errors in SpO_2_ readings caused by the pulse oximetry device, it can anticipate transient dips hence preventing unnecessary response to transient SpO_2_ dips that may occur for clinically insignificant reasons. This may have a beneficial effect on controlling the phenomena of alarm fatigue.

Importantly, SWIFT generalizes well across patient groups. We did not observe substantial differences in model performance between SWIFT-5 and SWIFT-30, nor between predictions made on ventilated vs. non-ventilated patients and COVID-19 vs. generally critically ill patients. The one exception was sensitivity, when aggregated across all timepoints–in the eICU test sets, more than 80% of hypoxemic events were detected as compared to more than 60% in the JH-CROWN test sets. This is unsurprising given that SWIFT was trained exclusively on non-COVID-19 patients, and the JH-CROWN database consists only of COVID-19 patients. Notably, the lung damage from SARS CoV-2 infection appears more severe than that from Acute Respiratory Distress Syndrome (ARDS) secondary to most other etiologies, and we are still in the early stages of understanding COVID-19 disease mechanistically. This difference in degree of lung damage may contribute to the performance differences.

Moreover, these predictions appear to be generalizable across hospitals and dates (the eICU database comprises patient-stays from 208 ICUs in 2014 and 2015, whereas the JH-CROWN database consists of patients from one medical center in 2020). Our test-sets contained male and female patients in roughly equal proportions, a range of admissions diagnoses, and substantial numbers of patients with non-white ethnicities (Table 1).

However, one limitation of SWIFT is that it was trained and tested primarily on older, critically ill patients. The median age of patients in each test-set was between 60 and 65 years old, and all data came from critically ill patients. Hypoxemia is a consideration in much younger patients as well, and future work will be needed to evaluate SWIFT-5 and SWIFT-30 on younger patients, or to train new models with additional data. A second limitation is that we did not train race-specific models. Recent work has shown that occult hypoxemia (low arterial oxygen saturation despite a pulse oximetry measurement between 92% and 96%) occurs far more frequently in Black patients than White patients [22]. For this reason, there is racial bias in interpretation of SpO_2_ values, which may not be well captured by our models (though our test sets are racially diverse; the JH-CROWN test sets have ~75% non-White patients). Regardless, SWIFT currently demonstrates high potential utility for simple, real-time prediction of hypoxemic events (occurrence and magnitude) 5 and 30 minutes in the future without the use of complex clinical informatics. As part of a clinical decision support system, SWIFT has the potential to inform the management of critically ill patients at risk for hypoxemia, including COVID-19 patients.

## Methods

### Data selection

First, we selected all patient ICU stays with mechanical ventilation at some point during the ICU stay from the eICU database (n = 1326) [13]. The eICU database consists of critically ill patients treated in 208 intensive care units across the United States in 2014 and 2015. We defined ICU stays with mechanical ventilation as distinct *patientUnitStayID* identifiers for which a respiratory chart entry included phrases similar to ET TUBE, ETT, Endotracheal, Trach, or Tracheostomy. Then, we randomly selected 1326 *patientUnitStayID* identifiers from those without indication of mechanical ventilation. We partitioned the first 1000 *patientUnitStayID* identifiers from the mechanical ventilation and no mechanical ventilation groups into a training set (n = 2000), and the last 326 from each group into two eICU test sets (eICU mechanical ventilation n = 326, eICU no mechanical ventilation n = 326). Then, we queried the vital signs time-series for each of these ICU stays, and excluded any ICU stays without corresponding SpO_2_ data recorded. This left 1933 stays in the training set, 326 stays in the mechanical ventilation test set, and 311 stays in the no mechanical ventilation test set. Since it is possible for a patient in the eICU database to have multiple ICU stays, we took the additional step of removing all ICU stays from the test sets for which that patient had a different ICU stay in the training set. This ensured that there was no overlap in patients between the train and test sets despite being unique ICU stays. This left 1933 stays (corresponding to 1859 patients) in the training set, 317 stays (corresponding to 285 patients) in the mechanical ventilation test set, and 311 stays (corresponding to 306 patients) in the no mechanical ventilation test set.

Second, all patient stays from the JH-CROWN database up to December 15, 2020 were selected (n = 301). The JH-CROWN database consists of COVID-19 patients seen in any Johns Hopkins Medical Institution facility with confirmed or suspected COVID-19. Each patient-stay in the JH-CROWN database corresponds to a unique patient (n = 301). Data extraction was performed using PostgreSQL, and the Python libraries psycopg2 and pandas [23].

Finally, those patients with entirely blank values for SpO_2_ were excluded. The eICU database contains vital signs recorded at 5 minute intervals, whereas the JH-CROWN database records vital signs at variable frequency. Therefore, the data in the JH-CROWN database was interpolated to 5 minute intervals by replacing blank values of SpO_2_ with the last valid observation. In the patients selected from the JH-CROWN database, the median time between observations was 25 minutes. If the first SpO_2_ value was missing, it was backfilled with the first available SpO_2_ value. Finally, those time series with less than 61 datapoints (5 hours) were excluded. This left 1837 patient-stays for model training, and 310, 288, and 298 patient-stays in the eICU Mechanical Ventilation, eICU No Mechanical Ventilation and JH-CROWN test sets respectively (S3 and S4 Figs).

### Data preparation

All SpO_2_ values were transformed using the following equation:

$$p=1-exp\left(\frac{SPO2-100}{10}\right)$$

This transformed value was chosen to magnify differences between SpO_2_ values close to 100%. Next, a causal moving average filter with a window of 5 was applied to each patient’s transformed SpO_2_ waveform (ie, the SpO_2_ values at the previous 4 timepoints and the current timepoint were averaged together. The first 4 available timepoints necessarily did not have smoothing applied). We chose this data smoothing technique since it is causal, meaning that it can be applied in real time, and it reduces transient noise hence providing a less noisy signal more suitable for clinical decision making. Other studies of hypoxemia prediction have also applied averaging filters to time-series data prior to prediction [6,9]. The time series for each patient was then down-sampled to 30 minute frequency for use with SWIFT-30 model which predicts SpO_2_ 30 minutes in the future. For SWIFT-5, which predicts SpO_2_ 5 minutes in the future, no changes were made.

Finally, the smoothed time series data for each patient was rearranged into an input vector X, and output vector Y where *p_n_* is SpO_2_ at timestep n:

$$X=\left[\begin{array}{c} p_{o}\;p_{1}\\ p_{1}\;p_{2}\\ .\\ .\\ .\\ p_{n-2}\;p_{n-1} \end{array}\right],Y=\left[\begin{array}{c} p_{2}\\ p_{3}\\ .\\ .\\ .\\ p_{n} \end{array}\right]$$

Finally, in the training set, all X vectors were concatenated and all Y vectors were concatenated to create one training set input vector and one training set output vector to be used in model training. In the 4 test sets, the patient-level input and output vectors were maintained to be used for model testing. Data preparation was performed in Python using standard data science libraries [23–25].

### Model training

A 3-fold cross validation procedure was used for hyperparameter optimization on the training data to evaluate 2 different LSTM model architectures (a deep architecture with 5 LSTM hidden layers and a shallow architecture with 2 LSTM hidden layers; both models had a Batch Normalization input layer and a Dense 1 neuron output layer and contained Dropout layers to prevent overfitting) and 3 different learning rates (ADAM optimizer with learning rates .001, .01 and .1). For both SWIFT-5 and SWIFT-30, the shallow architecture with learning rate .001 demonstrated the lowest average MSE across folds. For this selected architecture, the dropout ratio was .1. The first LSTM hidden layer had 256 nodes and the second had 16 nodes. This architecture was then used to re-train the final models on the full training set. The models were trained for 100 epochs with a random 10% validation set at each epoch. To prevent overfitting, the model weights at the epoch with lowest validation loss were used for the final SWIFT-5 and SWIFT-30 models. All model training was performed using the TensorFlow and Keras libraries in Python [26,27].

### Model testing

SWIFT-5 and SWIFT-30 were used to predict the transformed SpO_2_ waveform for each individual patient-stay in each of the three test sets. Then, the mean-squared-error and Pearson’s correlation coefficient were calculated between the true and predicted waveform for each patient-stay. Pearson’s correlation coefficient could not be calculated for 1 patient-stay in the JH-CROWN test set since the time series was constant and the correlation coefficient was undefined. Next, each time point was classified as hypoxemic or not based on a threshold of SpO_2_ 92% (transformed SpO_2_ .55067). Each prediction was also checked against the same threshold, and the sensitivity, specificity, accuracy, and PPV were calculated for each patient-stay time series. Sensitivity was not calculated for those patient-stays with no hypoxic events; Specificity was not calculated for those patient-stays with all hypoxic events; PPV was not calculated for those patient-stays for which no predictions were positive for hypoxemia, since these values are undefined in these cases. Finally, all timepoints in each test set were aggregated, and the false positive, true positive, false negative, and true negative rates were calculated for each test set.

## Supporting information

S1 Fig Examples of the best and worst fits by MSE for SWIFT-5 model tested on (a) eICU Ventilated patients–best fit, (b) eICU Ventilated patients–worst fit, (c) eICU Non-Ventilated patients–best fit, (d) eICU Non-Ventilated patients–worst fit, (e) JH-CROWN patients–best fit, (f) JH-CROWN patients–worst fit
(TIF)

S2 Fig Sensitivity and Specificity aggregated across all timepoints for each test-set, for SWIFT models using 1, 2, 3, 4 and 5 prior inputs. 2 prior inputs is the model architecture used for SWIFT-5 and SWIFT-30 presented in the paper. For SWIFT-5, 2 prior inputs corresponds to 10 minutes of prior data input, while for SWIFT-30, 2 prior inputs corresponds to 60 minutes of prior data.
(TIF)

S3 Fig Inclusion/Exclusion Diagram for patient-stays from eICU Database. (TIF)

S4 Fig Inclusion/Exclusion Diagram for patient-stays from JH-CROWN Database. (TIF)

S1 Table Analysis of differences in number of hypoxemic events between test-set patients classified with sensitivity less than .5 versus greater than or equal to .5. (DOCX)
