# Supplementary figures and images for: SWIFT: A deep learning approach to prediction of hypoxemic events in critically-Ill patients using SpO2 waveform prediction
Source: PLoS Comput Biol. 2021 Dec 21;17(12):e1009712. doi: 10.1371/journal.pcbi.1009712 (PMC8730462; doi:10.1371/journal.pcbi.1009712)

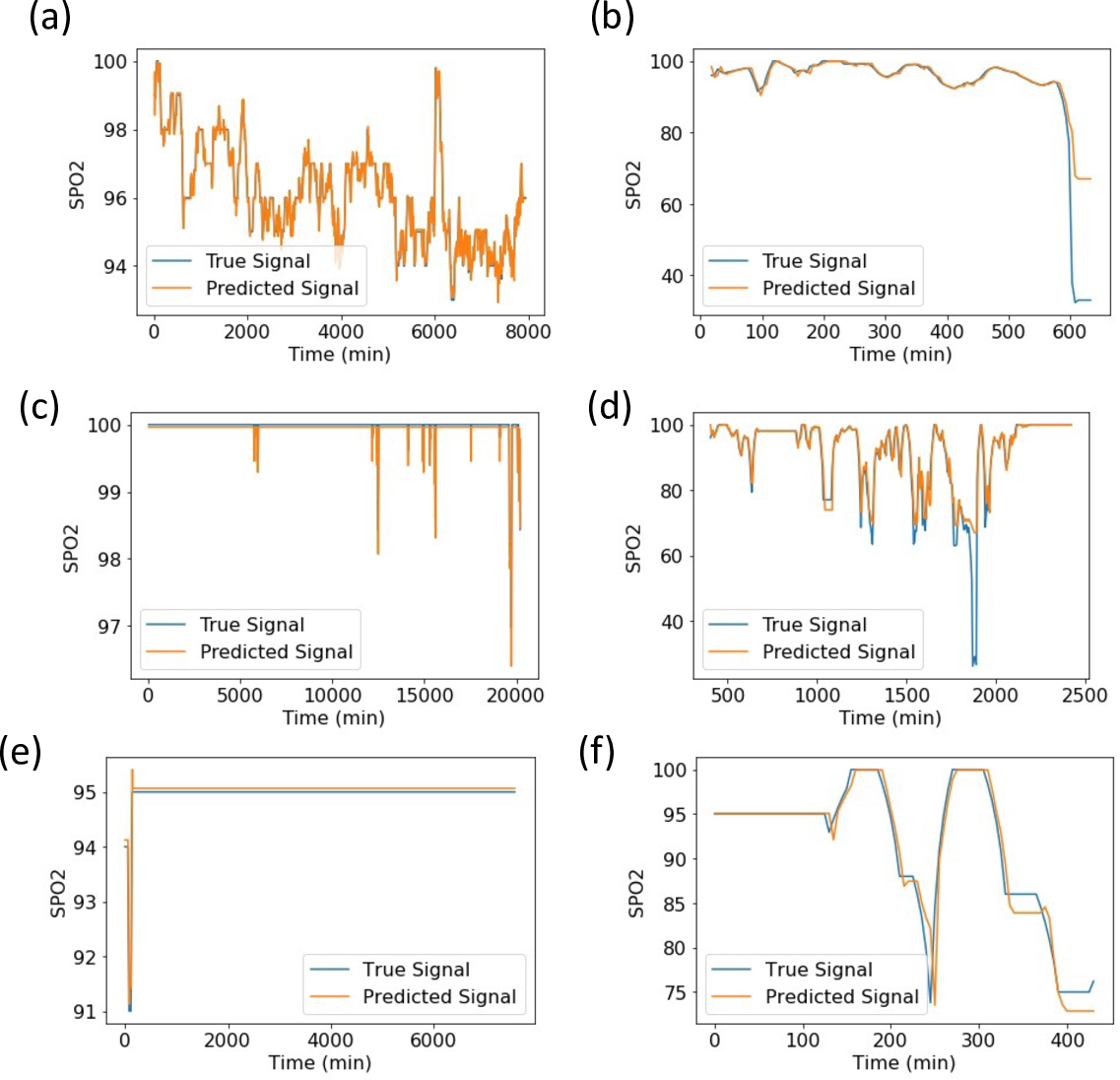

Supplement: S1 Fig — Examples of the best and worst fits by MSE for SWIFT-5 model tested on (a) eICU Ventilated patients–best fit, (b) eICU Ventilated patients–worst fit, (c) eICU Non-Ventilated patients–best fit, (d) eICU Non-Ventilated patients–worst fit, (e) JH-CROWN patients–best fit, (f) JH-CROWN patients–worst fit (TIF) [file pcbi.1009712.s001.tif]

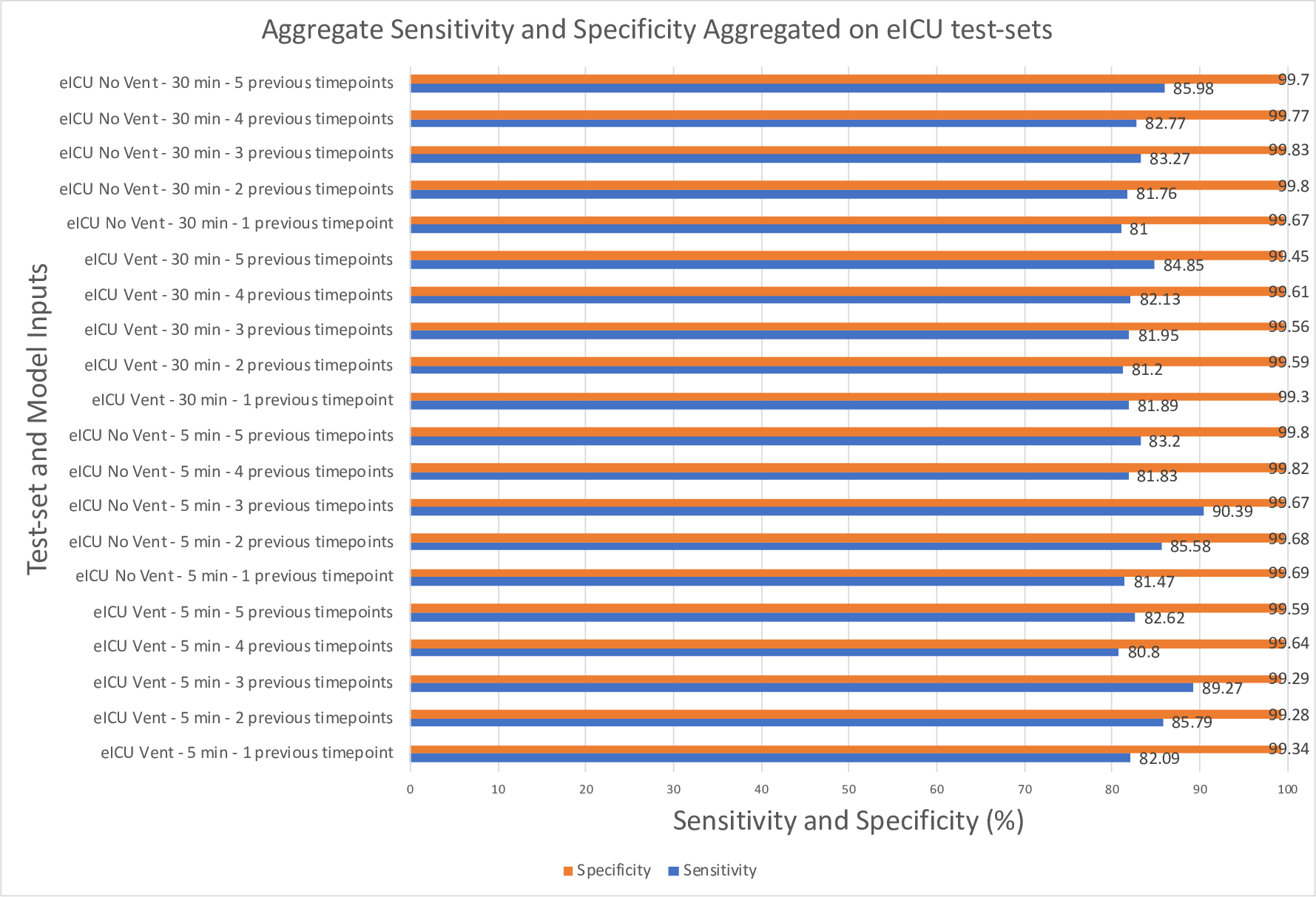

Supplement: S2 Fig — 2 prior inputs is the model architecture used for SWIFT-5 and SWIFT-30 presented in the paper. For SWIFT-5, 2 prior inputs corresponds to 10 minutes of prior data input, while for SWIFT-30, 2 prior inputs corresponds to 60 minutes of prior data. (TIF) [file pcbi.1009712.s002.tif]

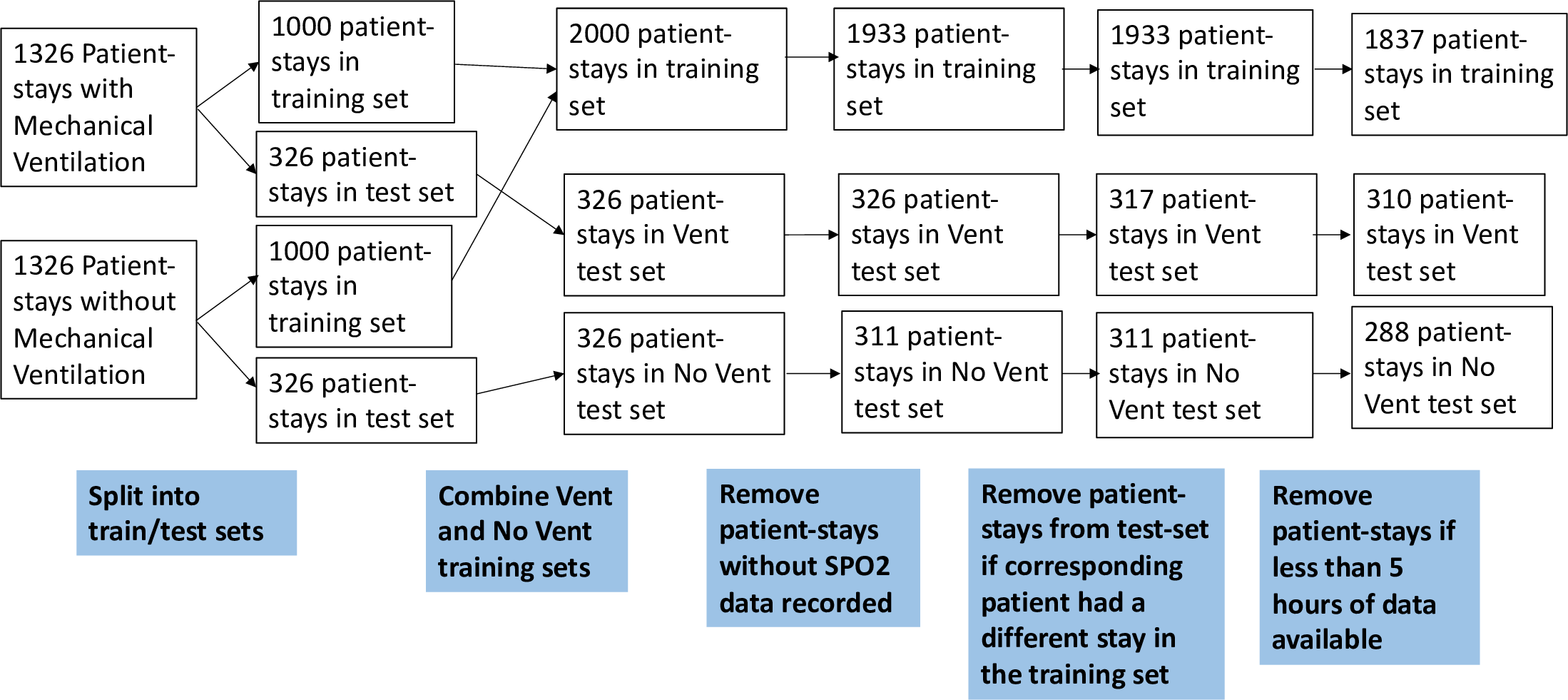

Supplement: S3 Fig — (TIF) [file pcbi.1009712.s003.tif]

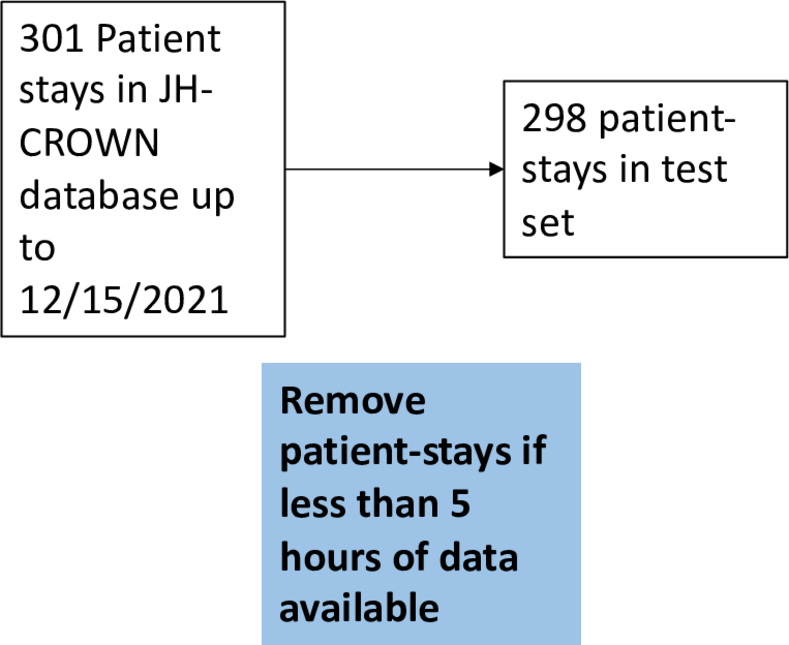

Supplement: S4 Fig — (TIF) [file pcbi.1009712.s004.tif]
