# Supplementary material for: SWIFT: A deep learning approach to prediction of hypoxemic events in critically-Ill patients using SpO2 waveform prediction
Source: PLoS Comput Biol. 2021 Dec 21;17(12):e1009712. doi: 10.1371/journal.pcbi.1009712 (PMC8730462; doi:10.1371/journal.pcbi.1009712)
Supplement: S1 Table — (DOCX) [file pcbi.1009712.s005.docx]

|  | **eICU Vent 30 min** | **eICU No Vent 30 min** | **eICU Vent 5 min** | **eICU No Vent 5 min** | **CROWN 30 min** | **CROWN 5 min** |
| --- | --- | --- | --- | --- | --- | --- |
| **Number of samples with sensitivity < .5** | **24** | **17** | **19** | **13** | **69** | **53** |
| **Number of samples with sensitivity > or = .5** | **222** | **133** | **238** | **152** | **219** | **235** |
| **Number of samples with no hypoxemic events** | **64** | **138** | **53** | **123** | **10** | **10** |
| **Median number of Hypoxemic events in samples with sensitivity < .5** | **3** | **2** | **7** | **5** | **61** | **367** |
| **Median number of Hypoxemic events in samples with sensitivity >= .5** | **15.5** | **9** | **85.5** | **40** | **92** | **560** |
| **Welch’s t-test for identical means between number of hypoxemic events in samples with sensitivity < .5 vs samples with sensitivity > or = .5** | **p = 1.71e-08** | **p = 9.14e-06** | **p = 6.54e-10** | **p = 1.13e-05** | **p = 2.62e-07** | **p = 1.07e-09)** |

**S1 Table: Analysis of differences in number of hypoxemic events between test-set patients classified with sensitivity less than .5 versus greater than or equal to .5**
